# EXPLORING EMOTION CONTROL BELIEFS ACROSS THE LIFESPAN: ROLES OF AGE AND CHRONIC ILLNESS

**DOI:** 10.1093/geroni/igad104.2160

**Published:** 2023-12-21

**Authors:** Jocelyn Rutledge, Meaghan Barlow

**Affiliations:** Wilfrid Laurier University, Waterloo, Ontario, Canada; Wilfrid Laurier University, Waterloo, Ontario, Canada

## Abstract

Existing theory and research highlights emotion control beliefs have important implications for emotion regulation and psychological well-being. Emotion control beliefs can be subdivided into four categories: the extent to which people believe 1) they can control their emotion (self-can beliefs), they should control their emotions (self-should beliefs), 3) others can control their emotions (others-can beliefs), and 4) other should control their emotions (others-should beliefs). To our knowledge, research has yet to examine how developmental factors are associated with emotion control beliefs in adults (i.e., age and non-normative experience of chronic illness). Past research highlights age related differences in emotional processes, and the influence of non-normative chronic illness on emotional well-being. Therefore, the present study utilized data from a lifespan sample of 47 participants (younger adults: n = 21, age range = 19-35, M = 27.55; older adults: n = 26, age range = 68-81, M = 72.40) to examine differences in emotion control beliefs among younger and older adults, people with and without chronic illness, and the interaction between these factors. Our analyses revealed levels of self-can beliefs were lower in people with chronic illness. In particular, younger adults with chronic illness reported the lowest levels of self-can beliefs. By contrast, among people without chronic illness, older adults reported lower levels of others-should beliefs, relative to younger adults. These findings inform theories of emotional aging, development, and health by suggesting emotion control beliefs vary with age and chronic illness.

